# Evolutionary Conservation of the Functional Modularity of Primate and Murine LINE-1 Elements

**DOI:** 10.1371/journal.pone.0019672

**Published:** 2011-05-10

**Authors:** Bradley J. Wagstaff, Miriam Barnerβoi, Astrid M. Roy-Engel

**Affiliations:** Tulane Cancer Center, Department of Epidemiology, Tulane University Health Sciences Center, New Orleans, Louisiana, United States of America; Tel Aviv University, Israel

## Abstract

LINE-1 (L1) retroelements emerged in mammalian genomes over 80 million years ago with a few dominant subfamilies amplifying over discrete time periods that led to distinct human and mouse L1 lineages. We evaluated the functional conservation of L1 sequences by comparing retrotransposition rates of chimeric human-rodent L1 constructs to their parental L1 counterparts. Although amino acid conservation varies from ∼35% to 63% for the L1 ORF1p and ORF2p, most human and mouse L1 sequences can be functionally exchanged. Replacing either ORF1 or ORF2 to create chimeric human-mouse L1 elements did not adversely affect retrotransposition. The mouse ORF2p retains retrotransposition-competency to support both Alu and L1 mobilization when any of the domain sequences we evaluated were substituted with human counterparts. However, the substitution of portions of the mouse cys-domain into the human ORF2p reduces both L1 retrotransposition and Alu *trans*-mobilization by 200–1000 fold. The observed loss of ORF2p function is independent of the endonuclease or reverse transcriptase activities of ORF2p and RNA interaction required for reverse transcription. In addition, the loss of function is physically separate from the cysteine-rich motif sequence previously shown to be required for RNP formation. Our data suggest an additional role of the less characterized carboxy-terminus of the L1 ORF2 protein by demonstrating that this domain, in addition to mediating RNP interaction(s), provides an independent and required function for the retroelement amplification process. Our experiments show a functional modularity of most of the LINE sequences. However, divergent evolution of interactions within L1 has led to non-reciprocal incompatibilities between human and mouse ORF2 cys-domain sequences.

## Introduction

The activity of LINE-1 (L1) elements has contributed both directly and indirectly to almost a third of the human genome mass [1]. Evidence of LINE retroelement activity dates as far back as 100 million years ago (mya) [1]–[3]. L1 continues to be active in the vast majority of mammalian species tested to date, with a few exceptions [4], [5]. Human and rodent lineages diverged approximately 80 million years ago, with each lineage harboring unique L1 subfamilies [6], [7]. Recent data demonstrate a significant contribution of L1 activity to human genomic diversity [8], [9] and somatic variation in human lung cancer genomes [10]. Current activity of L1 and its non-autonomous partners, Alu and SVA, account for about 0.3% of new human germ-line diseases [11]. Estimates suggest that retrotransposition occurs at a rate of one in 21, 212, and 916 births for Alu, L1, and SVA, respectively [12]. Since the split between eutherians and marsupials, a single L1 clade continues to amplify with separate single dominant lineages of L1 families in primates and rodents [13].

A full-length human L1 is about 6 kb, consisting of a 5′ untranslated region (UTR), two open reading frames separated by an intergenic sequence, and a 3′ UTR which ends in a poly(A) signal and an A-tail [14]. The two open reading frames, ORF1 and ORF2, code for activities necessary for L1 retrotransposition [15]–[17]. The general structure of these elements is relatively conserved throughout L1 evolutionary history. However, the 5′ UTR region containing the promoter sequence differs between L1 lineages of the same species [18], [19] and between human and rodent LINEs [20]. In addition, the ORF1 protein shows poor sequence conservation between human and rodent L1 subfamilies, particularly the amino terminus region (reviewed in [21]). The L1 ORF1 encodes a 40 kDa RNA binding protein which interacts with the L1 transcript to form a ribonucleoprotein (RNP) particle [22], [23]. Studies indicate that ORF1p functions as a chaperone [24] and is required for L1 retrotransposition [25]. The ORF2 encodes a 149 kDa protein with two known activities that can be assigned to specific domains. The N-terminus contains an endonuclease (endo) with sequence [17] and crystal structure [26] similar to the APE-1 endonuclease, a component of the base excision repair pathway. The reverse transcriptase (RT) activity is found in the central domain of ORF2p, flanked upstream by a conserved Z motif required for RT function [27]. Reverse transcription is critical, as mutations [16] and the addition of reverse transcriptase inhibitors suppress retrotransposition [28], [29]. The C-terminus or “cys-domain” contains a cysteine-rich motif (CX3CX7HX4C) that is essential for L1 retrotransposition [16]. Mutations within the conserved motif abolish the ability of ORF2p to interact with the L1 RNA [23]. However, the role of the rest of the cys-domain remains unknown. Further details on the role of the ORF2 protein are scarce due to the difficulty of its detection and the limited availability of robust ORF2p antibodies [30], [31]. Thus, researchers have used indirect methods to detect ORF2p by measuring the effects of its enzymatic processes [32]–[34] and developed alternate tools for its detection [23].

Comparison of the consensus sequences of the currently active young human and mouse L1 elements reveals different lineage-specific areas of amino acid conservation. In this manuscript, we present data from an array of chimeric human-mouse L1 elements and chimeric ORF2p that allowed us to determine species-specific differences that influence retrotransposition. In addition, these data provide insight into the less characterized cys-domain and suggest an important functional role for a less conserved region of the cys-domain that is distinct from the RNA binding function of the highly conserved cysteine-rich motif.

## Results

### Human LINE-1 retrotransposition competence and RNA primary sequence changes

**Figure 1:**
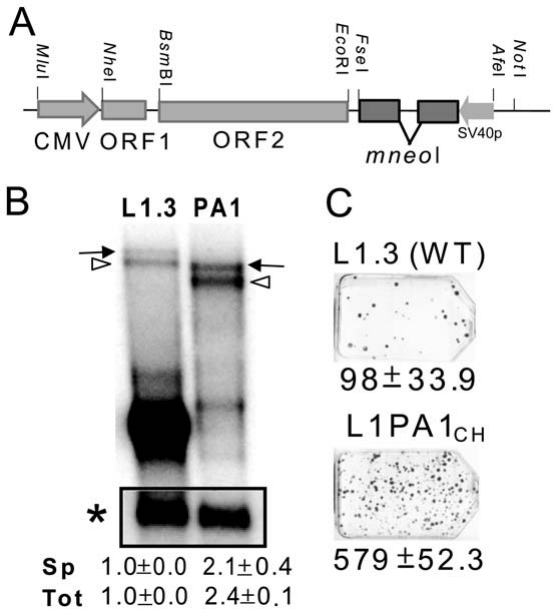
Codon optimized minimal human L1 is retrocompetent. **A**. Schematic of the optimized human L1 construct. The CMV promoter drives the transcription of the fully optimized ORF1 and ORF2 for L1_RP_. The L1 vector is tagged with the *mneoI* indicator cassette containing an inverted neomycin resistance gene. The SV40 promoter (SV40p) drives transcription of the neomycin gene which is disrupted by an intron in the opposite orientation that renders it non-functional [16]. The intron is only spliced out from tagged L1 transcripts generated by the CMV promoter. When retrotransposition of the spliced L1 RNA occurs, the new insert will contain a functional neo gene that is expressed from the SV40 promoter. Only retrotransposed copies of the spliced RNA will confer G418 resistance. Some of the unique restriction sites used in the construction of the other vectors are shown. **B**. Northern blot analysis of the RNA profiles, 48 hours post-transfection, of transiently transfected HeLa cells with the tagged “wild type” L1.3 construct JM101/L1.3 (L1.3) and the tagged optimized minimal human L1PA1_CH_ *mneo* (PA1) using a probe to the 3′ region of the neomycin gene. The full-length unspliced tagged L1 transcript (arrow) and the transcript with spliced neo tag (open arrowhead) are indicated. The inset shows the cyclophilin transcript (asterisk). The spliced (Sp) full length L1transcript and the total (spliced and unspliced; Tot) full length L1 RNA were normalized to cyclophilin and calculated relative to the L1.3 construct (designated as 1.0). The mean ± the standard deviation for the quantitation results for each construct is indicated below (n = 2). The highly expressed low molecular weight band observed for L1.3 likely represents common splice transcript variants that exclude most or all of the L1 coding sequences previously shown to be produced by L1 elements [36]. **C**. The retrotransposition capability of L1PA1_CH_ and L1.3 was assayed in HeLa cells. The optimized minimal human L1 is retrotransposition competent.

LINE-1 elements from different species (human, mouse and zebrafish) contain multiple premature polyadenylation signals and splice donor and acceptor sites within their sequences [35]–[37], leading to the generation of a diverse array of processed transcripts. Because RNA processing and secondary structure could possibly affect retrotransposition rate, we chose to minimize the impact of the differences between the L1 sequences contributing to effects on RNA levels by working with codon optimized human and mouse coding sequences driven by the CMV promoter (see methods). Thus, our approach should largely confine any species-specific observations to the amino acid sequences of the human and mouse L1 elements. Moreover, past studies utilizing synthetic codon optimized L1 constructs improved transcription of the full length retrocompetent L1 transcripts, increased protein expression and augmented retrotransposition rates in *ex vivo* assays [33], [38], [39]. These observations suggest that the RNA largely plays a passive role within the L1 RNP as a retrotransposition intermediate. Our codon optimized version of a minimal L1_RP_ (pBS-L1PA1_CH_ *mneo*, Figure 1) is consistent with these observations. We find that optimization reduces some of the processing of the full length L1 RNA and that our tagged minimal human L1 construct generates about twice as much full length L1 mRNA compared to the wild-type L1.3 element (transcripts with the spliced or unspliced neo tag) (Figure 1B). The highly expressed low molecular weight band observed for L1.3 in Figure 1B likely represents common splice transcript variants that exclude most or all of the L1 coding sequences previously shown to be generated by L1 elements [36]. Transient transfection assays reveal that the optimized minimal L1 also retrotransposes with high efficiency compared to wild-type L1 (Figure 1C).

### Human and mouse ORF1 and ORF2 are interchangeable

**Figure 2:**
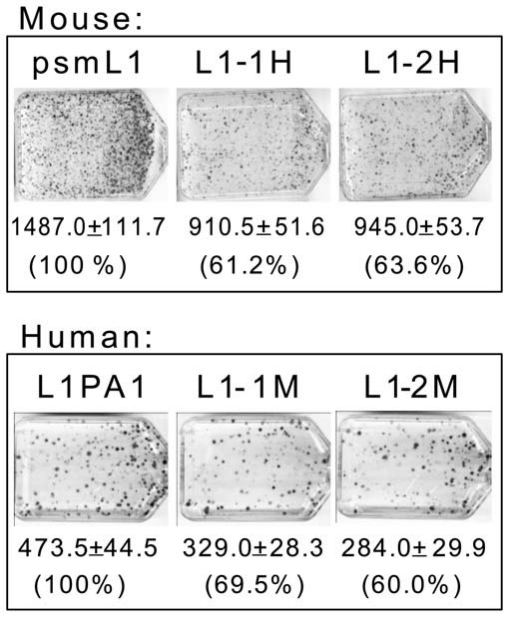
Mouse and Human ORF1 and ORF2 are interchangeable. Retrotransposition assay results of tagged mouse (psmL1), human (pBS-L1PA1_CH_ *mneo*), and chimeric ORF1–2 L1 constructs. Human-mouse ORF1–2 chimeras were made from the parental constructs of both species by replacing either the ORF1 or ORF2 with the equivalent ORF from the other species. Each set of three flasks shows the parental single species (left), chimeras with swapped ORF1 (middle), and chimeras with swapped ORF2 (right). Average number of colonies and standard deviation from two replicate experiments is shown for each construct. ^a^Percent activity relative to the parental is indicated for each of the chimeras.

The human and rodent L1 lineages have evolved independently for approximately 80 million years. The ORF1p and ORF2p of the currently active human and mouse elements share approximately 35% and 63% amino acid identity, respectively. To test for a species-specific interaction between L1 ORF1–ORF2 proteins, we individually substituted the ORF1 or ORF2 sequences of a tagged mouse (psmL1) and human (L1PA1) L1 construct with the same ORF from the opposite species and used these chimeric L1 elements in the retrotransposition assay. All of the chimeric ORF1–ORF2 L1 constructs maintain retrocompetence (Figure 2); however, the chimeric L1 retrotransposition rates were lower than the parental counterparts, ranging between 60–69.5% activity relative to the parental elements.

### Generation of human-mouse ORF2 chimera

**Figure 3:**
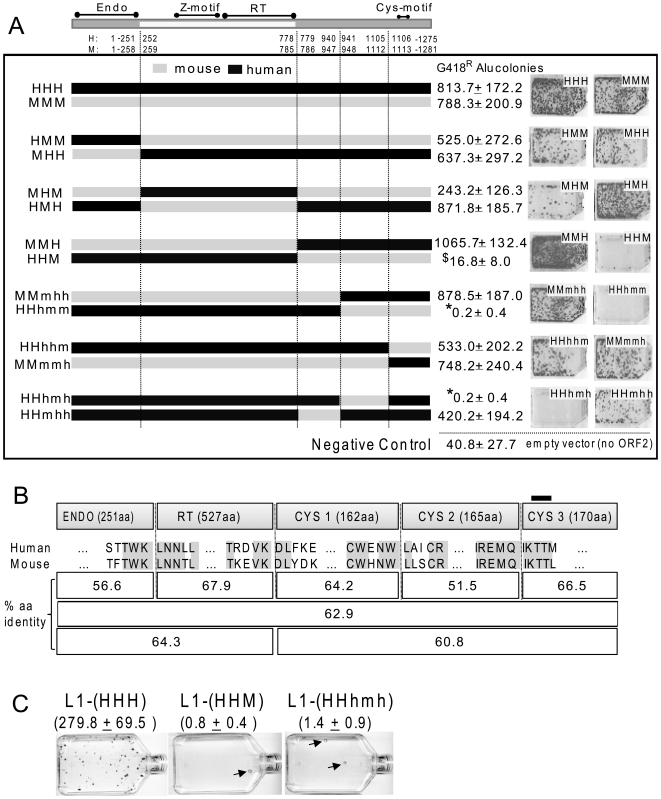
L1 ORF2p human-mouse chimera. **A. Chimera breakpoint schematic of chimeric ORF2s and their ability to drive Alu retrotransposition** ***in trans*** **.** A schematic for the L1 ORF2 protein with its three recognized domains, endonuclease (endo), reverse transcriptase (RT) and the ill-defined 3′ region containing the cysteine-rich motif (cys), subdivided into three parts. The amino acid positions flanking the selected break points are indicated for human and mouse ORF2s. The constructs are named using 3 capital letters, each representing a domain: H for human and M for the mouse portions, where HHH and MMM represent complete human and mouse ORF2 proteins, respectively. In constructs where the cys-domain was further subdivided, the third capital H or M was replaced with three lowercase h or m letters, indicating the subdivision of the cys-domain. HeLa cells were co-transfected with a tagged Alu and the different chimeric ORF2 expression constructs. Results from the Alu retrotransposition assay average colony counts with standard deviation, along with images of representative colony assays, are shown to the right of each of the construct schematics. Averages are derived from six replicate experiments. Assay results using an empty vector (*i.e.*, no ORF2 supplied in *trans*) show the average number of background colonies of Alu retrotransposition events that are inferred to be derived from endogenously expressed ORF2p in human HeLa cells. An asterisk (*) represents significant difference from empty vector, p<0.01, two-tailed two sample T-test; $ represents not significantly different from empty vector. **B. Chimera breakpoints and amino acid conservation between human and mouse L1 ORF2 proteins.** ORF2p chimera breakpoints and numbers of amino acids (aa, taken from the human ORF2p) are shown in the first row. The next two rows show the five amino acid residues on each side of the breakpoints (ORF2s from human L1_RP_ and mouse L1_spa_). Conserved human and mouse residues are highlighted. The three bottom rows show average amino acid conservation between human and mouse proteins for the indicated ORF2p segments. The location of the cys-motif is shown at the top as a dark bar. **C. Tagged LI constructs with substituted chimeric ORF2p HHM and HHhmh in place of the parental ORF2 significantly limit L1** ***cis*** **retrotransposition capability.** L1 retrotransposition assay results in HeLa cells using the parental human L1 construct (pBS-L1PA1_CH_ *mneo*, shown as L1-HHH) and the same construct with substituted chimeric ORF2, as indicated. The observed G418^R^ foci (mean ± standard deviation) are indicated for three repeats of the experiment, with the L1 chimeras showing significantly fewer colonies than the parental L1 (200–350 fold difference; p<0.0001, one-tailed two sample T-test). A representative of the retrotransposition results is shown with arrows indicating infrequent colonies from the chimeric L1 transfections.

We generated 12 human-mouse ORF2 chimeric proteins by swapping homologous sequences between species and selected sequence breakpoints to preserve distinct functional domains (Figure 3A). We subdivided the multifunctional L1 ORF2 protein into its three previously described regions: the endonuclease (EN), the reverse transcriptase (RT) plus the Z-motif, and the carboxy-terminus domain containing the cysteine-rich motif (cys). The cys domain was further subdivided into three stretches of roughly equal size. The chimera breakpoints with their relation to the ORF2 domains and the 12 chimeras are detailed in Figure 3A. The endonuclease containing region is well characterized with a clearly defined self-sufficient domain [26], [40]. We defined the RT domain region as the sequence starting immediately 3′ of the EN domain, including the required Z-motif, and terminating with the RT domain as recognized by the conserved domain database (cdd pfam00078.12) from NCBI [41]. We refer to the remaining carboxy-terminus ORF2p residues, including the cys-motif at residues 1129–1147, as the “cys domain.” Because the function of the cys domain is largely unknown, we further subdivided this region into three segments of roughly equal size for more detailed analysis, with the carboxy-terminal segment containing the conserved cys-motif.

Chimera breakpoints were introduced in areas of conserved amino acid sequence ranging from 3–9 identical residues surrounding the breakpoints (Figure 3B). The protein alignment is unambiguous with only a single seven residue insertion/deletion difference between human and mouse ORF2p at the beginning of the endo domain. Thus, the breakpoints within the ORF2 of each species are certain to be at identical positions. Complete alignments of the ORF2p from several mammalian species, including human and mouse, demonstrate high conservation of the amino acid sequence at the site of the breakpoints (Figure S1). Amino acid conservation of the individual ORF2p sections varies (Figure 3B). There is clearly some heterogeneity of divergence, where the endo region (56.6%) and the middle cys 2 region (51.5%) show lower amino acid identity than the complete protein (62.9%).

### The mouse and human ORF2 endonuclease and reverse transcriptase domains are interchangeable

We evaluated the functionality of the chimeric ORF2 proteins by assessing their ability to support Alu retrotransposition *in trans* in the human HeLa cell line. Because ORF2p alone is sufficient to drive Alu retrotransposition in the assay [42], we did not use ORF1 in any of the Alu retrotransposition experiments. This approach simplified the interpretation of our ORF2 chimera results, since an additional complicating variable (human or mouse ORF1) was not required to assess functionality. The four reciprocal chimeras with swapped endonuclease (mouse with human endo: HMM; and human with mouse endo: MHH) and RT domains (mouse with human RT: MHM; and human with mouse RT: HMH) were all functional, with only MHM showing more than a 35% drop in retrocompetence (30.9% activity relative to mouse: MMM) (Figure 3A).

### Sequences from the cys-domain of mouse ORF2 are incompatible with the human protein

#### Inefficient support of Alu retrotransposition in trans

The mouse ORF2p is functional when any portion of its cys domain is substituted with human counterparts (Figure 3A: mouse with the human cys: MMH and MMmhh, and MMmmh). The same is not true for the human ORF2 protein. The only human chimera with a portion of the mouse cys domain that effectively supported Alu retrotransposition *in trans* was HHhhm (Figure 3A). The other ORF2 construct variants with different portions of the mouse cys sequences in the human ORF2p (HHM, HHhmm, and HHhmh) did not support Alu retrotransposition. Transfections with HHhmm and HHhmh chimeras produced significantly fewer colonies than the negative empty vector control (two-tailed, two sample T-test, p<0.01) and HHM transfections showed a similar trend but were not significantly different than empty vector. We therefore considered that the colonies from these three sets of transfections likely represent background colonies derived from endogenous L1 proteins in HeLa cells [43] and that the means below background level indicate either a dominant negative effect or toxicity associated with ORF2 transfections [44], [45]. Even if these chimera function at a very low efficiency (*i.e.*, ∼1000 fold reduction), a decrease in retrotransposition rate of this magnitude compares to that observed for the endonuclease and reverse transcriptase mutant L1 constructs, which are considered to be essentially “non-functional” [16]. Transfection of these constructs in the mouse NIH3T3 cell line yielded comparable results (Figure S2) with proportionally fewer overall colonies. It is not surprising to observe lower numbers as retrotransposition assay efficiency varies across cell lines and even between different laboratory stocks of the same cell line [46]. The three ORF2 chimeras (HHM, HHhmm, and HHhmh) were unable to support Alu retrotransposition (*i.e.*, not significantly different from the empty vector control; two sample T-test, p≥0.238) irrespective of the cell line utilized, while HHhhm was not significantly different from the single species ORF2p positive control (two sample T-test, p = 0.21).

#### Inefficient support of L1 retrotransposition

We selected two of the “non-functional” ORF2p chimera (HHM and HHhmh) for evaluation in the context of our tagged human L1 construct. As with *trans*-mobilization of Alu, we observed between one and two L1 colonies per experiment (Figure 3C), suggesting that these two chimeric ORF2p are effectively “non-functional” at supporting L1 retrotransposition in *cis*. The introduction of these cys-domain mouse sequences into the ORF2p of the human L1 element reduces retrotransposition by 200–350 fold.

#### Evaluating expression and protein functions of the ORF2 human-mouse chimera

**Figure 4:**
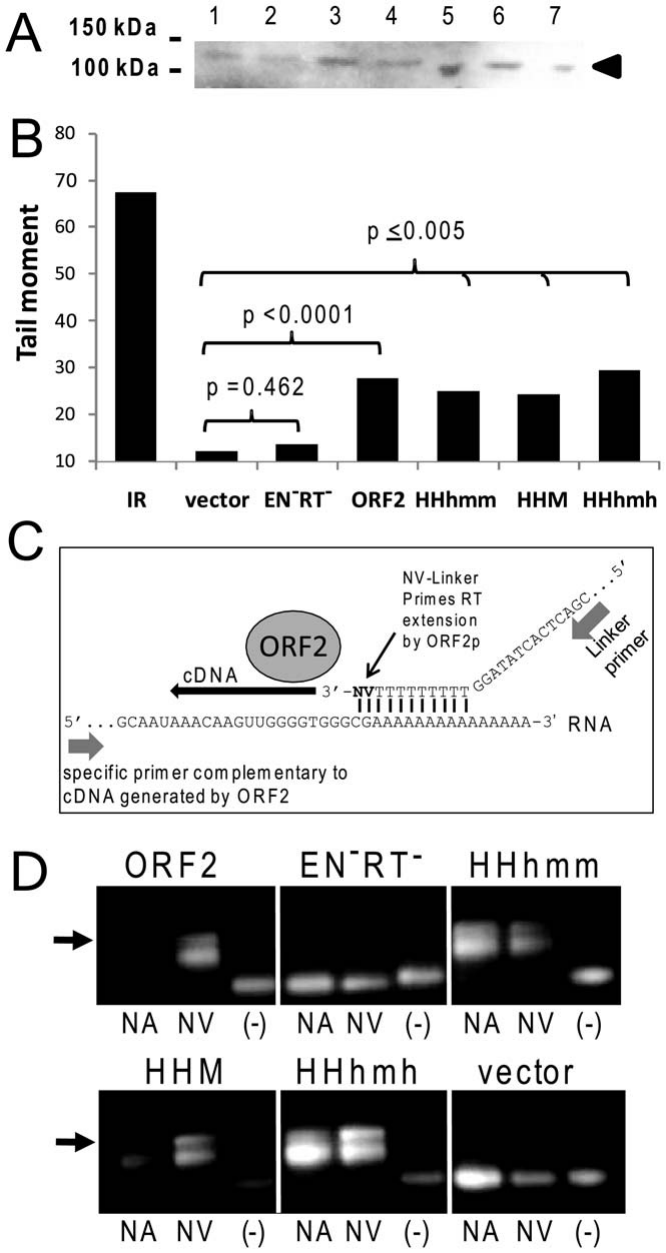
Evaluation of chimeric ORF2p expression and functionality. **A. Detection of ORF2 protein.** Western blot analysis of extracts from transiently transfected HeLa cells with constructs expressing the following human-mouse ORF2 chimeric proteins: 1- MMmhh, 2- HHhmm, 3- HHM, 4- MMH, 5- HHhmh, 6- HHmhh, and 7- optimized human ORF2 (HHH). An arrow indicates the expected position of the 149 kDa ORF2 protein. **B. Evaluation of endonuclease activity.** Alkaline comet assay for the detection of DNA breaks of transiently transfected cells plasmids expressing the optimized human ORF2 (HHH), an endonuclease and reverse transcriptase mutant (EN^−^RT^−^), and the three chimera that are incapable of inducing retrotransposition above background levels (HHM, HHhmm, and HHhmh). The experiment is one representative of three repetitions. Results from Student paired T-test relative to the empty plasmid control (vector) are indicated. Significant differences were observed for all ORF2 constructs, except for the RT^−^ and EN^−^ ORF2 mutant (negative control). Note that all cells are exposed for the gamma-irradiated control (IR), while only a portion of the cells transfected with the ORF2 constructs express the ORF2 protein of interest due to the transfection efficiency. **C. Schematic of the LEAP assay.** The LEAP assay was previously developed for the detection of L1 ribonucleoprotein (RNP) complexes containing ORF2p and an RNA template [47]. The assay consists of a partial purification of a cytoplasmic subcellular fraction containing ORF2p and L1 RNA as part of the L1 RNP. Isolated RNPs, along with nucleotides and a linker oligo (to function as the priming site), were used for *in vitro* detection of the reverse transcriptase activity of the L1 ORF2p [23]. Two types of linkers are used: one with “no anchor” having only thymidine residues at the 3′ end (NA; not shown) and one with two residues to function as an anchor at the 3′ end (NV; shown). The generated cDNA is detected by PCR amplification using a primer complementary to the cDNA and to the sequence of the linker oligo. **D. Evaluation of reverse transcriptase activity.** Reverse transcriptase activity of the different ORF2 chimera was assessed with the LEAP assay using primers to detect cDNA generated from the ORF2 transcript. Extracts from HeLa cells transfected with the different ORF2 chimeric constructs were assayed using the linker with no anchor (NA) or the linker with anchor (NV). No linker oligo was added to the negative control (−). The empty plasmid (vector) and the RT^−^ and EN^−^ ORF2 mutant were used as negative controls. The arrow indicates the expected size product for a positive result.

Inefficient expression could explain the reduced retrotransposition activity observed for the ORF2 chimeric protein. We assessed protein expression using western blot analysis of extracts from cells transfected with all the chimeric ORF2 constructs. A band with an apparent molecular weight (∼150 kDa) corresponding to ORF2p was detected for human and chimeric ORF2p transfections (Figure 4A). These results suggest that all of the ORF2 chimera constructs are capable of generating stable levels of protein.

The lack of the actual structure of the complete ORF2 protein limits the predictions on how changes in the cys-domain of these chimeric ORF2 proteins may affect the endonuclease or reverse transcriptase activities. Therefore, we proceeded to assess whether the presence of mouse cys-domain sequences in the human ORF2p might affect the two known activities of the protein. We evaluated the endonuclease activity of the chimeric ORF2 proteins showing poor retrotransposition capability using the alkaline COMET assay that detects both single strand nicks and double strand breaks introduced in DNA. All of the tested ORF2p chimera generated a significantly higher number of DNA nicks/breaks (Student paired T-test, p≤0.005) compared to the empty vector and the EN^−^RT^−^ ORF2p control (Figure 4B). We next assessed reverse transcriptase activity using the previously described LEAP assay that was developed for L1 [47] and shown to be useful for detecting activity from cell extracts transfected with constructs that express ORF2p alone [23]. This assay detects ORF2p-RNA (RNP) complexes by evaluating the ability of ORF2p to reverse transcribe the RNA *in vitro* (detailed in Figure 4C). Following the published protocol, we utilized the two described linker oligos to generate the cDNA. One contains a poly-adenine stretch at the 3′ end, referred to as “no anchor (NA),” and the other contains a poly-adenine stretch that ends with an anchor (NV; see Figure 4C). Our results indicate that the retrotransposition-deficient ORF2p chimera maintain an active reverse transcriptase that can still interact effectively with its RNA (Figure 4C). Overall, these data indicate that the inactivity of the HHhmm, HHM and HHhmh ORF2p chimera appear to be due to an undetermined but critical function of the cys-domain.

## Discussion

L1 elements have been replicating and evolving in mammals for approximately 100 million years. Because a retroelement is generally parasitic upon its host genome, the potential for parasite-host coevolution may have contributed to some independent evolution of L1 sequences and host factors in different lineages. The basic organizational structure of L1 is the same for all mammalian elements, consisting of the 5′UTR-ORF1–ORF2-3′UTR and poly(A). Previous *in vivo* work demonstrated that the L1 5′UTR can be completely removed and replaced by another unrelated promoter, such as the composite CMV enhancer modified chicken β-actin promoter [48]. In addition, the complete removal of the 3′UTR sequence still allows for L1 retrotransposition. Although the 3′UTR contains a conserved G-rich motif (G) [49], data suggest that the 3′UTR is not essential for the retrotransposition process but may instead provide a supportive role. Codon optimization of L1 sequences could alter L1 RNA folding and affect its interaction with ORF1 or ORF2. However, as previously observed for the mouse L1spa [38] and human L1_RP_ [33], [39], these changes do not negatively impact L1 activity. Our minimal optimized L1 is retrotranspositionally more efficient than the wild type tagged L1.3. Several factors likely contribute to this result, including a higher inherent L1_RP_ retrotransposition rate relative to L1.3, an increased amount of the full length L1 transcript, and possibly an increase in translation efficiency due to the codon optimization of the construct.

The modularity of L1 sequences becomes more evident with our observation that chimeric L1 human-mouse ORF1–ORF2 constructs maintain retrotransposition competence. Of particular interest, the ORF1 sequence was subjected to intense positive selection during a brief period of primate evolution, resulting in a high rate of amino acid replacement in the coiled-coil domain, which is currently conserved in the human ORF1p [50]. The same ORF1 region in rodent L1 lineages also experienced a high rate of amino acid replacement [3], [51]–[53]. The coiled-coil domain mediates ORF1p multimerization and it has been suggested that it may also promote interaction with other proteins, possibly ORF2p [54], [55]. Recent data demonstrate that mutations in the putative leucine zipper or RRM of ORF1p lead to reduced amounts of ORF2p in L1 RNP containing fractions [23]. However, our data suggest that although there might be important species-specific epistatic interactions between ORF1p and ORF2p, these inter-protein interactions are not sufficient to abolish the retrotransposition competence of our chimeric constructs.

The ability of chimeric human-mouse ORF2 proteins to mobilize Alu *in trans* was independent of the species (mouse or human) of the transfected cell line. We previously demonstrated that ORF2p from both mouse and human L1 sources mobilizes tagged Alu transcripts in chicken cells [56]. Our observations suggest that the potential interaction of ORF2p chimera with species-specific host factors is not required in our experimental assays. Amino acid comparison of the human and mouse ORF2 proteins identifies two regions with decreased conservation relative to the complete protein (Figure 3B). The two regions are the endonuclease with 56.6% identity and the middle subsection of the cys-domain with 51.5% identity (Figure 3B, details in Figure S3). Unlike the cys-domain, the endonuclease domain was easily exchanged between the two species. We anticipated the endonuclease modularity, as a similar approach demonstrated the interchangeability of endonuclease domains for the non-LTR elements SART1 and TRAS1 [57]. In addition, previous *in vitro* work demonstrated that this domain maintains endonuclease activity independent of the rest of the ORF2 protein [17], [58]. The reverse transcriptase domain also proved to be interchangeable. The RT domain contains ten highly conserved regions that are shared amongst most non-LTR retrotransposons [59]. This high conzservation probably reflects functional constraints on the reverse transcriptase and is consistent with our observation that mouse and human RT sequences are interchangeable.

The simplest interpretation of our data is that predominantly human ORF2 protein is incompatible with the middle subsection of the mouse cys domain. All three of our chimeras that fit this description were essentially non-functional, while reciprocal mouse ORF2 chimeras maintained retrotransposition competence. Functionality of these chimeras could not be rescued by the use of cells from either species or by the addition of ORF1p in the context of the bicistronic L1 constructs. Given the important role that the cys-domain appears to play in functionality, it is notable that the highly conserved cys-motif is not associated with the observed species-specific difference. Reciprocal chimeras that only affected the third subsection of the cys-domain containing the conserved cys-motif (HHhhm and MMmmh) were both highly functional.

The inhibitory effect caused by one or more sequence stretches of the mouse cys-domain in the context of the human protein appears to be independent of the endonuclease or reverse transcriptase activities of ORF2p, as demonstrated by our functionality assays. Previous work demonstrated that substitutions of the cysteines in the cysteine rich motif lowered the amount of ORF2p in the RNP fraction and reduced LEAP activity, suggesting a possible reduction in the ORF2p-RNA interaction [23]. In contrast, our retro-incompetent ORF2p cys-domain chimera (HHhmm, HHM and HHhmh) show no reduction in the LEAP assay. Our observations reinforce separate roles for the cys-motif and the middle section of the cys-domain. However, it remains possible that the exchanged region has an interface with the endonuclease and/or RT domain in the retroincompetent ORF2p chimera. Our data show that retrocompetence can be recovered from the non-functional HHM by swapping the human endonuclease domain for the mouse (*i.e.*, HHM→MHM). However, the MHM ORF2 is only ∼30% active relative to the parental HHH or MMM constructs. We observe a higher recovery effect with the RT domain, as functionality is also restored when the non-functional HHM is compared to the functional HMM construct with ∼66% activity of HHH or MMM. Because activity is not fully restored in either of the two chimeric proteins, it is possible that the decrease in retrocompetence is due to negative sequence interactions and/or inefficient structural folding of the ORF2 protein.

Interestingly, the smallest tested sequence with negative impact (165 aa of the middle sub-section of the cys-domain) is also the one with the lowest amino acid identity (Figure 3B), which could possibly indicate a region that has evolved in response to interactions with other L1 sequences or host factors. Without more detailed knowledge of the structure and function of the cys-domain of ORF2p, any interpretations at this time are limited. Our data show that a region of the cys-domain has evolved to lose some of the modularity that is preserved by the other ORF2 domains. Phylogenetic analysis of the timeframe of cys-domain evolution could show whether or not it is correlated with amino acid changes in other L1 coding regions and provide insight into the nature of the sequence and/or protein interactions that influence chimera functionality.

## Materials and Methods

### Constructs

pBS-L1PA1_CH_ *mneo*, contains the fully codon optimized ORF1 and ORF2 of the human L1_RP_ cloned in the pBluescriptII vector (Stratagene). A schematic of the synthetic L1 vector is shown in Figure 1A. The L1 open reading frames were codon optimized using Primo Optimum 3.4 (http://www.changbioscience.com/primo/primoo.html), which makes synonymous changes to optimal codons within the nucleotide sequence. A 63 bp inter-ORF with 92% sequence identity to L1_RP_ was utilized for all constructs. Sequence changes to this region were generated to introduce restriction enzyme sites used for plasmid construction. The optimized nucleotide sequence is 74.9% identical to the sequence of L1_RP_. The “notag” constructs contain an SV40 polyadenylation signal at the 3′ end, and the others contain the *mneo*I cassette including the SV40 polyadenylation signal from JM101/L1.3 [60].

pBS-L1PA1_CH_ *rescue*, contains the L1_RP_ ColE1-mneo cassette of SynL1neo [61] instead of the *mneoI* cassette. The ColE1-mneo cassette was amplified with the FseI-NeoTag: 5′-TCCTAGCTGGCCGGCCTTTTATTGCCGATCCCCT-3′ and Not-NeoTag: 5′ –TCCTAGCTGCGGCCGCAACGCGCGAGGCAGCCGGATCATA-3′ and cloned into the *Fse*I and *Not*I sites of pBS-L1PA1_CH_ *mneo* (Figure 1).

pBudORF2 human-mouse chimera were generated by a PCR approach using overlapping primers [62] using pBudORF2opt [33] and psmL1“ORFeus” derived from L1_spa_ (a kind gift from Dr. Jef Boeke) [38] as source sequences. ORF2opt codes for the L1_RP_ ORF2 protein sequence and was codon optimized by Blue Heron Biotechnology, Inc (Bothell, WA). The open reading frames are cloned into the expression vector pBudCE4.1 (Invitrogen), under control of the CMV promoter. The nomenclature and break points of the chimeric ORF2 constructs are described in Figure 3A.

pBS-L1-1H,2M*mneo* and pBS-L1-1M,2H*mneo* were derived from pBS-L1PA1_CH_ *mneo* and contain either the mouse ORF1 or ORF2 of psmL1 [38] as source DNA.

pCEP-L1synM-1H and pCEP-L1synM-2H contain the codon optimized human ORF1 or ORF2 (L1_RP_) in psmL1 [38].

pBS-L1-1H,2HHM *mneo*, and pBS-L1-1H,2HHhmh *mneo* were derived from pBS-L1PA1_CH_ *mneo* and contain the indicated human-mouse chimeric ORF2 as shown in Figure 3A.

JM101/L1.3, referred to as “wildtype” L1, contains a full-length copy of the L1.3 element and the *mneo*I indicator cassette cloned in pCEP4 (Invitrogen) [60], [63].

All plasmid DNA was purified by alkaline lysis and twice purified by cesium chloride buoyant density centrifugation and quality assessed by the visualization of agarose gel electrophoresed aliquots stained with ethidium bromide. Constructs were confirmed by sequencing (Elim Biopharmaceuticals Inc, Hayward,CA).

### L1 and Alu retrotransposition Assays

Transient L1 or Alu retrotransposition assays were performed as previously described with some minor modifications [34]. Briefly, HeLa (ATCC CCL2) or NIH3T3 (ATCC CRL1658) cells were seeded at a density of 5×10^5^/T75 flask or 2.5×10^5^ cells/T25 flask. Transient transfections were performed the following day with Lipofectamine Plus (Invitrogen) following the manufacturer's protocol using 1 µg of Alu-*neo* ^TET^ vector plus 0.33 µg of the ORF2 construct or empty vector (T25 flask). For L1 retrotransposition assays between 1 or 3 µg of the tagged L1 plasmids was used for transfection (T75 flask). The next day, the cells were treated with the appropriate media containing 400 µg/ml for HeLa and 800 µg/ml for NIH3T3 cells of Geneticin/G418 (Fisher Scientific). After 14 days, cells were fixed and stained for at least 30 minutes with crystal violet (0.2% crystal violet in 5% acetic acid and 2.5% isopropanol). Retrotransposition rate was then determined as the number of visible G418^R^-resistant colonies/10^6^ cells seeded. Both L1 and Alu G418 resistant colonies were validated as authentic retrotransposition events (Figure S4).

### Northern blot analysis

HeLa cells were seeded at a density of 4×10^6^ for transfection with 5 µg of the L1 plasmid constructs. Cells were harvested 48 hours post-transfection for RNA extraction. RNA extraction and poly(A) selection was performed as previously described [35]. The polyadenylated transcripts were electrophoresed in a 1% agarose-formaldehyde gel and then transferred to a Hybond-N nylon membrane (Amersham Biosciences). The RNA was UV cross-linked to the membrane using a UV-light (GS Gene linker, BioRad). The membrane was pre-incubated in hybridization solution: 30% formamide, 1× Denhardt's solution, 1% SDS, 1 M NaCl, 100 µg/ml salmon sperm DNA, 100 µg/ml yeast t-RNA at 60°C for at least 3 hr. A riboprobe to the 3′ region of the neomycin gene was generated using a PCR product amplified with the following primers T7neo(−): 5′-TAATACGACTCACTATAAGGACGAGGCAGCG-3′ and Neo northern(+): 5″- GAAGAACTCGTCAAGAAGG-3′. The cyclophilin riboprobe was generated from pTRI-cyclophilin template (Ambion) to use as a control for transcript quantitation analyses. Riboprobes were radioactively labeled by incorporating ^32^P-UTP (Amersham Biosciences) using the MAXIscript T7 kit (Ambion) following the manufacturer's recommended protocol. The radiolabeled probes were purified by filtration through NucAway Spin columns (Ambion). Separate hybridizations were performed overnight with 4–12×10^6^ cpm/ml of each individual probe at 60°C. The membrane was washed twice at high stringency (0.1× SSC, 0.1%SDS) at 60°C before analysis using a Typhoon Phosphorimager (Amersham Biosciences) and the ImageQuant software.

### Western blot analysis

Protein was extracted from trypsinized and PBS-washed transiently transfected NIH3T3 or HeLa cells using standard Tris SDS glycerol buffer plus 2-mercaptoethanol and boiled for 15 min. Extracts were electrophoresed on 4–12% NuPage Bis-Tris gels (Invitrogen). Proteins were transferred to a nitrocellulose membrane using the iBlot gel transfer system (Invitrogen). The goat anti-LINE (S-19, an affinity purified goat polyclonal antibody raised against a peptide mapping near the ORF2 N-terminus of human LINE-1) and secondary HRP-conjugated antibodies were purchased from Santa Cruz Biotechnology Inc. The membrane was incubated for 1 h at room temperature with the primary or secondary antibody diluted 1∶500 and 1∶5000 in PBS pH 7.4 plus 0.05% Tween 20 and 3% non-fat dry milk (Biorad), respectively. Signals were detected using the SuperSignalWest Pico Chemiluminescent Substrate (Pierce, Rockford, IL) and Amersham ECL hyperfilm (GE Healthcare).

### Comet assay

HeLa cells were transfected with 1 µg of plasmid expressing the different ORF2 expression plasmids and controls. After 24 hours, cells were harvested and gently resuspended in phosphate buffer saline (PBS pH 7.4) to a concentration of 10^5^ cells/ml. Gamma-irradiated (5 Gy) cells were used as positive control, untreated cells as background and the empty vector plus an endonuclease and reverse transcriptase ORF2 mutant as negative controls. The alkaline comet assay was performed by using the CometAssay Reagent Kit for Single Cell Gel Electrophoresis (Trevigen, Gaithersburg, MD) following the manufacturer's instructions. Cells were mixed with low melting agarose (Comet LMAgarose, Trevigen) and embedded on a slide (CometSlide, Trevigen). Slides were incubated in cold lysis solution (Trevigen) for 45 minutes at 4°C. Slides were then incubated in Alkaline Solution, pH>13 (300 mM NaOH, 1 mM EDTA) for 40 minutes at room temperature in the dark. Cells were then electrophoresed in Alkaline Electrophoresis Solution, pH>13 (300 mM NaOH, 1 mM EDTA) in a horizontal gel apparatus (Bio-Rad, Hercules, CA) at 1 V/cm for 30 minutes at 4°C. Slides were rinsed briefly in dH_2_O and then incubated in 70% ethanol for 5 minutes. Slides were air-dried overnight and DNA stained with SYBR Green I (Trevigen). Cells were analyzed using an epifluorescence microscope (Nikon, Melville, NY). Digital images were acquired with a SensiCam QE digital camera (Cooke Corporation, Romulus, MI). Tail moments were determined for at least 75 random cells per sample by using Comet Assay IV (Perceptive Instruments, United Kingdom) comet scoring software. Tail moment is defined as the ratio of DNA in the comet tail to total DNA.

### ORF2 LEAP assay

HeLa cells (4×10^6^/T75 flask) were transfected with 10 µg of the ORF2-expressing constructs. Cells from two transfected flasks were pooled and harvested 48 h post-transfection following the previously described protocol [47]. The LEAP reaction product was generated using the published linkers, LEAP NV: 5′-GAGCACAGAATTAATACGACTCACTATAGGTTTTTTTTTTTTVN-3′ and LEAP NA: 5′-GCGAGCACAGAATTAATACGACTCACTATAGGTTTTTTTTTTTT-3′. No LEAP primer was added to the negative control. The LEAP cDNA product was PCR amplified using the anchor primer (5′- GCGAGCACAGAATTAATACGACT-3′) and a primer specific to a sequence shared by both the mouse and human ORF2p constructs near the 3′end of the gene (ORF2-3′B: 5′-ACGGATCCGAACAAAAACTCATC-3′). A 3 µl aliquot of the LEAP product was PCR amplified for 35 cycles of 20 s at 94°C, 30 s at 62°C and 30 s at 72°C, with a final cycle of 5 min at 72°C and visualized on a 2.5% agarose gel.

## Supporting Information

Figure S1 **Amino acid conservation between mammalian L1 ORF2 proteins.** We used the Clustal W algorithm of the MegAlign alignment program (LaserGene; DNAstar) to align ORF2 protein sequences from human (L1RP), mouse (L1spa), rat, dog, rabbit, cow, tree shrew, and horse. Sequences other than human and mouse were collected from the Repbase Update database [1]. The consensus is shown at the top; amino acid residues shaded gray are identical to the human ORF2 sequence. The four breakpoints used in chimera construction follow position numbers 268, 795, 957, and 1123 from this specific alignment.
(TIF)

Figure S2 **The human and mouse chimeric HHM, HHhmm, and HHhmh chimeric L1 ORF2ps are unable to support Alu retrotransposition in a rodent cell line.** NIH3T3 (mouse) cell line was transiently transfected with the three ORF2p chimeric proteins that were non-functional in HeLa and a tagged Alu vector. All three constructs (HHM, HHhmm, and HHhmh) generated Alu colonies at a rate that was not significantly different than the empty vector control. The results in rodent cells NIH3T3 emulate the results obtained in the human HeLa cells, the other cys-domain mutant (HHhhm) was functional. The functionality of these ORF2 human-mouse chimera appears to be independent of cell line species (mouse or human). Two sample T-test analysis showed: * Significantly different than HHM, HHM2, HHhmh, and empty vector p<0.01; $ Not significantly different than empty vector p≥0.238; # not significantly different than MMM p = 0.210.
(TIF)

Figure S3 **Amino acid alignment between the non-exchangeable region of the L1 ORF2 cys domain of mouse and human shows low and dispersed amino acid identity.** The region encompassing 160 amino acids of the mid-section of the cys domain of the L1 ORF2p is shown. The consensus is shown at the top; identical amino acids are shown and non-identical amino are shaded in gray.
(TIF)

Figure S4 **The L1 and Alu inserts recovered present the features of** ***bona fide*** **retrotransposed elements.** L1 and Alu G418^R^ colonies were selected and grown to confluency. DNA was extracted from the cells using the DNA Easy kit (Qiagen) following the manufacturer's recommended protocol. **A**. L1 inserts were recovered and analyzed using a previously published method [2]. The representation of two examples of recovered inserts generated by L1PA1_CH_ tagged with the mneoI/ColE1 cassette [3] is shown. Both examples shown are 5′ truncated L1 sequences (3088 bp and 3099 bp excluding the Atail). The tagged L1 sequence is represented as “L1+ColE1-mneo cassette” highlighted in gray. The tandem site duplications are underlined. The canonical polyadenylation signal is underlined and shown in italics. **B**. The extracted DNA from cells transfected with the tagged Alu vector plus the different ORF2 expression constructs was evaluated by performing nested PCR with primers designed to amplify the sequences flanking the self splicing intron, F1: 5′-GGGCGCCTGTAGTCCCAGCTA -3′; F2: 5′-TAGCAGCCAGTCCCTTCCCGCTTCA-3′; R1: 5′-GTCAGCGCAGGGGCGCCCGGTTC-3′ and R2: 5′-ACTGGGCACAACAGACAATCGGC-3′ ). The annealing location of the primers are shown in the schematic of the Alu construct. An open arrowhead indicates the PCR product corresponding to an insert containing the spliced version (open arrowhead) of the Alu expression vector. An open arrowhead indicates the nested 217 bp PCR product corresponding to an insert containing the spliced version (open arrowhead) of the Alu expression vector. Lanes correspond to the following chimeric ORF2 constructs: 1- MMmhh, 2- MMH, 3-HHmhh, 4-HHhhm, 5- MMmmh, 6- HMM, 7- MHH, 8- MHM, and 9- HMH. M denotes the DNA, and the Alu expression plasmid (P lane) was used as the control for 625 bp unspliced product (black arrow).
(TIF)
