# Supplementary figures and images for: Evolutionary Conservation of the Functional Modularity of Primate and Murine LINE-1 Elements
Source: PLoS One. 2011 May 10;6(5):e19672. doi: 10.1371/journal.pone.0019672 (PMC3091869; doi:10.1371/journal.pone.0019672)

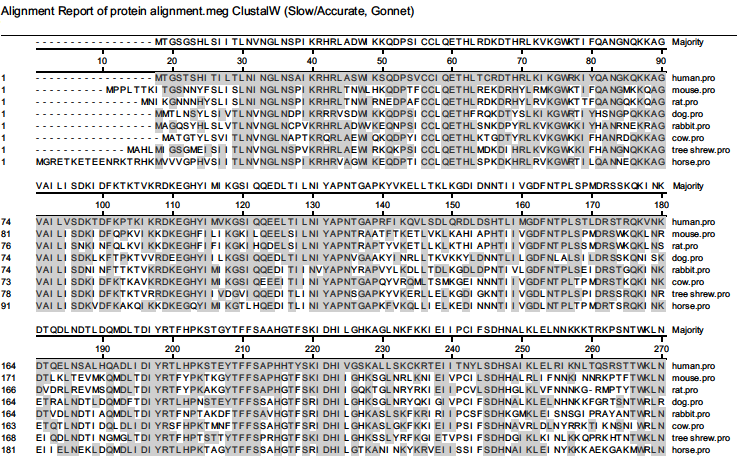

Supplement: Figure S1 — Amino acid conservation between mammalian L1 ORF2 proteins. We used the Clustal W algorithm of the MegAlign alignment program (LaserGene; DNAstar) to align ORF2 protein sequences from human (L1RP), mouse (L1spa), rat, dog, rabbit, cow, tree shrew, and horse. Sequences other than human and mouse were collected from the Repbase Update database [1]. The consensus is shown at the top; amino acid residues shaded gray are identical to the human ORF2 sequence. The four breakpoints used in chimera construction follow position numbers 268, 795, 957, and 1123 from this specific alignment. (TIF) [file pone.0019672.s001.tif]

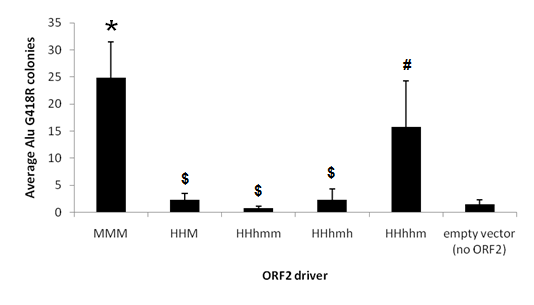

Supplement: Figure S2 — The human and mouse chimeric HHM, HHhmm, and HHhmh chimeric L1 ORF2ps are unable to support Alu retrotransposition in a rodent cell line. NIH3T3 (mouse) cell line was transiently transfected with the three ORF2p chimeric proteins that were non-functional in HeLa and a tagged Alu vector. All three constructs (HHM, HHhmm, and HHhmh) generated Alu colonies at a rate that was not significantly different than the empty vector control. The results in rodent cells NIH3T3 emulate the results obtained in the human HeLa cells, the other cys-domain mutant (HHhhm) was functional. The functionality of these ORF2 human-mouse chimera appears to be independent of cell line species (mouse or human). Two sample T-test analysis showed: * Significantly different than HHM, HHM2, HHhmh, and empty vector p<0.01; $ Not significantly different than empty vector p≥0.238; # not significantly different than MMM p = 0.210. (TIF) [file pone.0019672.s002.tif]

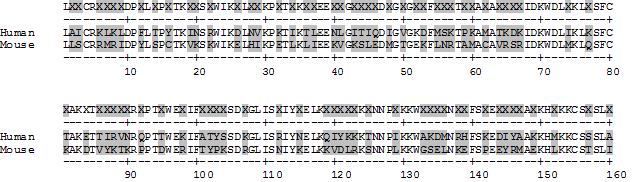

Supplement: Figure S3 — Amino acid alignment between the non-exchangeable region of the L1 ORF2 cys domain of mouse and human shows low and dispersed amino acid identity. The region encompassing 160 amino acids of the mid-section of the cys domain of the L1 ORF2p is shown. The consensus is shown at the top; identical amino acids are shown and non-identical amino are shaded in gray. (TIF) [file pone.0019672.s003.tif]

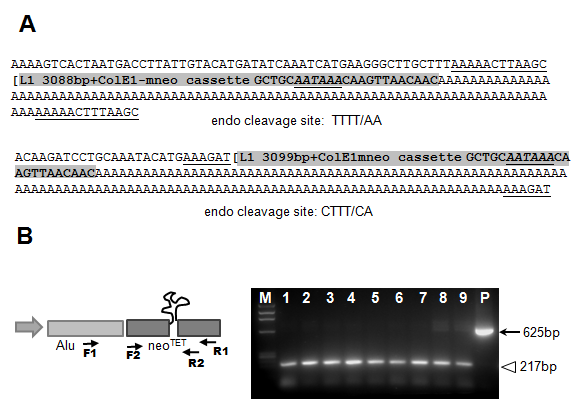

Supplement: Figure S4 — The L1 and Alu inserts recovered present the features of bona fide retrotransposed elements. L1 and Alu G418R colonies were selected and grown to confluency. DNA was extracted from the cells using the DNA Easy kit (Qiagen) following the manufacturer's recommended protocol. A. L1 inserts were recovered and analyzed using a previously published method [2]. The representation of two examples of recovered inserts generated by L1PA1CH tagged with the mneoI/ColE1 cassette [3] is shown. Both examples shown are 5′ truncated L1 sequences (3088 bp and 3099 bp excluding the Atail). The tagged L1 sequence is represented as “L1+ColE1-mneo cassette” highlighted in gray. The tandem site duplications are underlined. The canonical polyadenylation signal is underlined and shown in italics. B. The extracted DNA from cells transfected with the tagged Alu vector plus the different ORF2 expression constructs was evaluated by performing nested PCR with primers designed to amplify the sequences flanking the self splicing intron, F1: 5′-GGGCGCCTGTAGTCCCAGCTA -3′; F2: 5′-TAGCAGCCAGTCCCTTCCCGCTTCA-3′; R1: 5′-GTCAGCGCAGGGGCGCCCGGTTC-3′ and R2: 5′-ACTGGGCACAACAGACAATCGGC-3′ ). The annealing location of the primers are shown in the schematic of the Alu construct. An open arrowhead indicates the PCR product corresponding to an insert containing the spliced version (open arrowhead) of the Alu expression vector. An open arrowhead indicates the nested 217 bp PCR product corresponding to an insert containing the spliced version (open arrowhead) of the Alu expression vector. Lanes correspond to the following chimeric ORF2 constructs: 1- MMmhh, 2- MMH, 3-HHmhh, 4-HHhhm, 5- MMmmh, 6- HMM, 7- MHH, 8- MHM, and 9- HMH. M denotes the DNA, and the Alu expression plasmid (P lane) was used as the control for 625 bp unspliced product (black arrow). (TIF) [file pone.0019672.s004.tif]
